# Soybean images dataset for caterpillar and *Diabrotica speciosa* pest detection and classification

**DOI:** 10.1016/j.dib.2021.107756

**Published:** 2021-12-31

**Authors:** Maria Eloisa Mignoni, Aislan Honorato, Rafael Kunst, Rodrigo Righi, Angélica Massuquetti

**Affiliations:** a Universidade do Estado de Mato Grosso Carlos Alberto Reyes Maldonado, Avenida das Garças, 1192, Centro, Nova Mutum, MT 78450-000, Brazil; b Centro Universitário Univag, Av. Dom Orlando Chaves, 2655, Bairro Cristo Rei, Várzea Grande, MT 78118-900, Brazil; c Applied Computing Graduate Program University of Vale do Rio dos Sinos (Unisinos), Av. Unisinos, 950, Bairro Cristo Rei, São Leopoldo, RS 93022-750, Brazil; d Graduate Program in Economics University of Vale do Rio dos Sinos (Unisinos), Av. Dr. Nilo Peçanha, 1600 - Boa Vista, Porto Alegre, RS 91330-002, Brazil

**Keywords:** Agriculture, Soybean, Images datasets, Insects

## Abstract

This article presents a dataset of insect-damaged soybean leaves. The capture of images was carried out on several soy farms, under realistic weather conditions, using two cell phones and a UAV. The dataset consists of 3 (three) folders with a total of 6,410 images. The dataset is divided into three categories: (I) healthy plants, (II) plants affected by caterpillars, and (III) images of plants damaged by *Diabrotica speciosa*. This dataset allows training and validation of machine learning models to diagnose, recognize, and classify soybeans affected by caterpillars or *Diabrotica speciosa*. The images can be processed according to the user’s need since only the size was standardized during the pre-processing phase.

**Specifications Table**

**Table:** 

Subject	Computer Science, Agricultural Science, Biological Science
Specific subject area	image processing
Type of data	Raw
How data were acquired	The images were captured with 3 (three) different pieces of equipment, a Unmanned aerial vehicle (UAV), and two smartphones equipped with a 48mp AI triple camera.
Data format	The images are in JPEG format with a standard size of 500 × 500 pixels.
Description for data collection	Photographs were shot on sunny, windy, and cloudy days. The images focus on the upper part of the insect-infected soy leaves and healthy leaves.
Data source location	All photos were captured in the State of Mato Grosso, Brazil. More specifically, we chose two locations: (I) farms located in the municipalities of Lucas do Rio Verde - Latitude 13${}^{\circ }$ 01′ 59″ longitude 55${}^{\circ }$ 56′ 38″ and (II) farms located in the municipality of Nova Mutum - Latitude 13 ${}^{\circ }$ 05′ 04″, longitude 56${}^{\circ }$ 05′ 16″.
Data accessibility	The images are available online on Mendeley website. Repository name: Images of Soybean Leaves Direct link to the dataset: https://data.mendeley.com/datasets/bycbh73438/1 Data identification number (permanent identifier, i.e. DOI number): https://doi.org/10.17632/bycbh73438.1

## Value of the Data

• The data provide images of soybean leaves collected with smartphones and an UAV under variable weather conditions;
• Researchers belonging to different areas can benefit from this dataset. Computer scientists and data scientists can benefit from the provided data for training and evaluating machine learning and deep learning models for various purposes, like analyzing, recognizing, and diagnosing pests. Agricultural Engineers can use the dataset and the models generated based on the dataset to deal with potential diseases in the initial stages, reducing their impact and, consequently, maximizing production;
• This dataset can potentially impact society since it allows the creation of both classification and prediction models to improve food production by reducing the impact of pests in soybean crops. Images dataset and artificial intelligence algorithms optimize the analysis process, positively impacting the productivity of agricultural commodities, such as soybeans. Automated diagnosis is essential to prevent and control diseases in soybean and minimize economic losses [1].

## 1 Data Description

The dataset comprises three folders with a total of 6410 images. The first folder contains images of healthy plants. The second one stores photographs of plants affected by caterpillars. Finally, images of plants damaged by *Diabrotica speciosa* are in the last folder. Three experts conducted the selection and annotation of the images that compose the dataset.

Table 1 describes the type of damage/leaf condition, pests, and the number of images per folder.

**Table 1:** Types of damage and amount of sheet damage images.

Folder	Number of images
Healthy	896
Caterpillar	3309
*Diabrotica speciosa*	2205

**Total number of images**	**6410**

## 2 Types of Pests in the Dataset

**Fig. 1:**
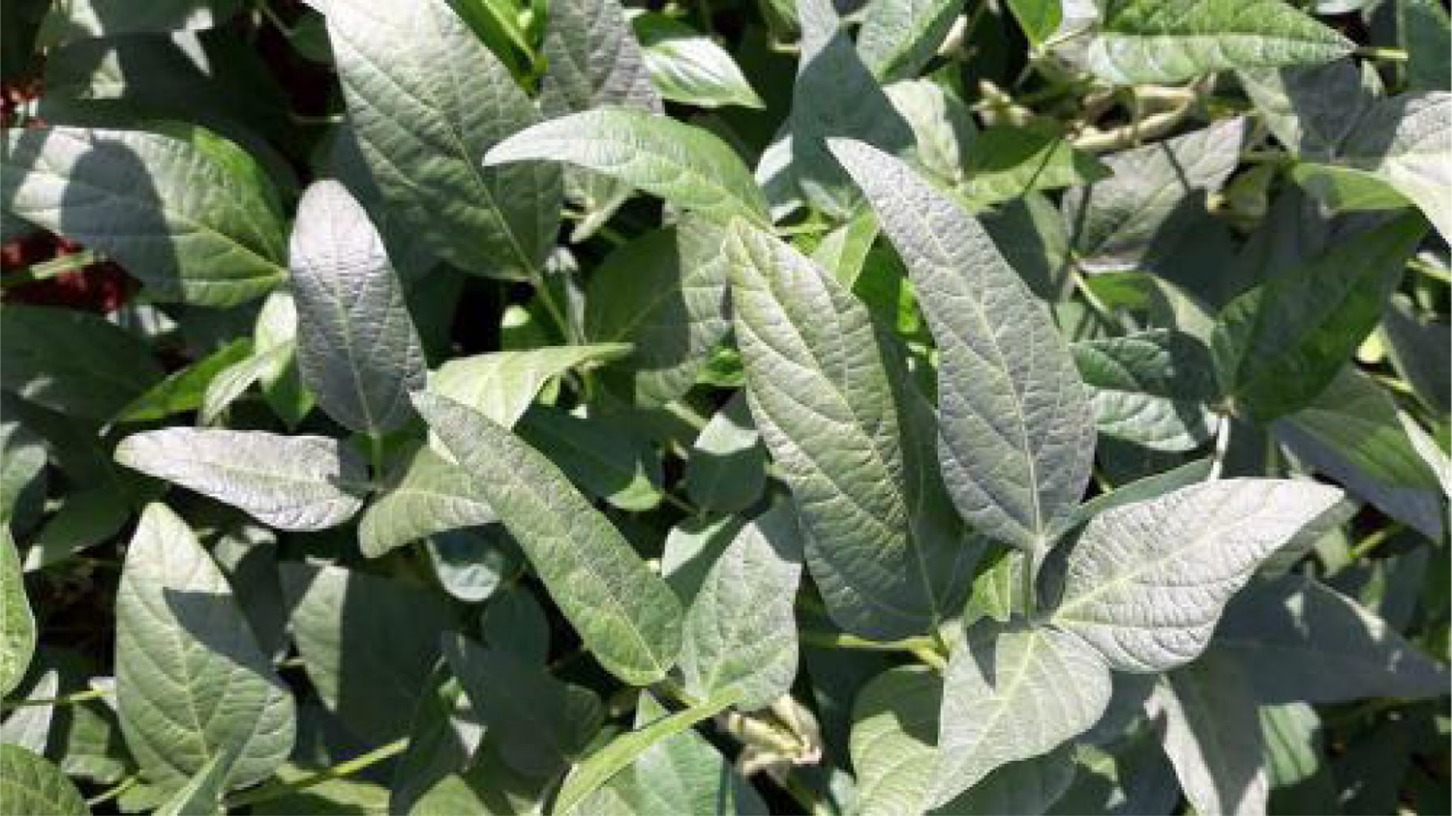
Healthy soy leaves.

In this section, we present examples of healthy leaves, as shown in Fig. 1. The dataset also comprises plants affected by two types of pests: caterpillars and *Diabrotica Speciosa* of the *Chrysomelidae* family.

### 2.1 Caterpillars

Caterpillars are pests that attack leaves, stems, pods, and grains, depending on their gender. In some cases, they can attack more than one item. The most common types of caterpillars that attack the leaves found in soybean crops are *Anticarsia gemmatalis* (Soybean caterpillar), *Chrysodeixis includens* (False measuring caterpillar), *Spodoptera* (Cartridge caterpillar), and *Omiodes indicalus* (Rolling caterpillar)), [3]. The *Spodoptera* caterpillars initially scrape the surface of the leaves and then start to devour mainly pods and grains.

**Fig. 2:**
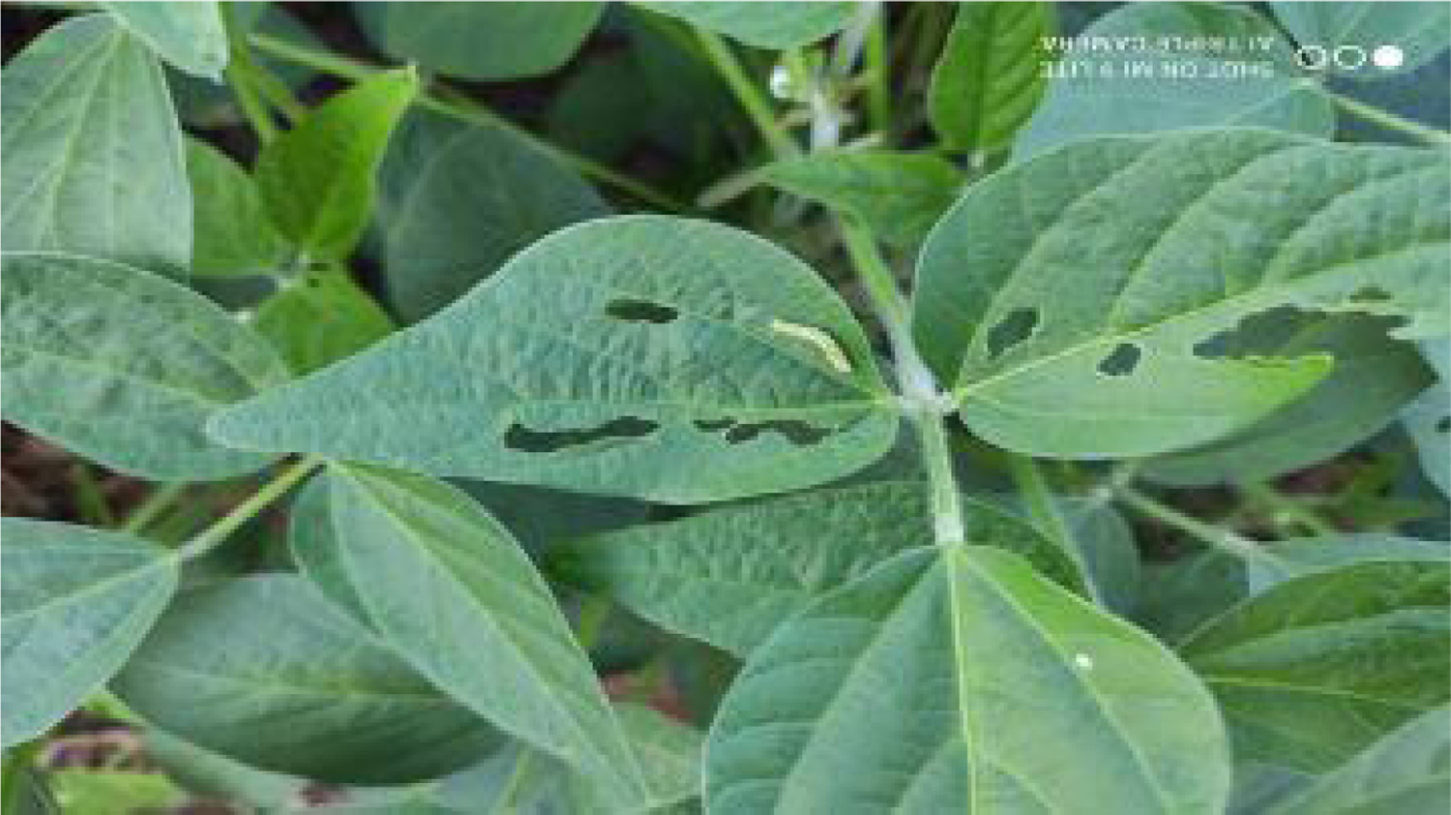
Soy leaves demaged by caterpillars.

In the dataset, the one with the highest incidence is *Spodoptera* caterpillars, more than 90%. The caterpillars of the Spodoptera complex are *S. frugiperda, S. cosmioides, S. eridania*, and *S. albula*. They make up a significant group of pests that attack soybean pods [7]. Caterpillars of the *Spodoptera* genus can cause damage to seedlings, reducing the growing stand, but they have also been reported as defoliators in the reproductive stage and feed on pods. Fig. 2 shows leaves damaged by caterpillars. Caterpillar damage has the shape of tears eaten from the sides to the center.

### 2.2 Diabrotica speciosa

Among the species of the *Chrysomelidae* family found in the soybean crop in the region where the images were collected, the most common is *Diabrotica speciosa*, commonly called green cow or patriot. This kind of pest prefers the softer leaves. When feeding, they make small round holes in the leaf. *Diabrotica speciosa* also makes incisions on the edges of the leaves [4]. *Diabrotica speciosa* in the adult stage are defoliators and can cause direct damage to pods and flowers [2]. Images 3 and 4 show samples of the damage caused to soybean leaves by *Diabrotica speciosa*.

**Fig. 3:**
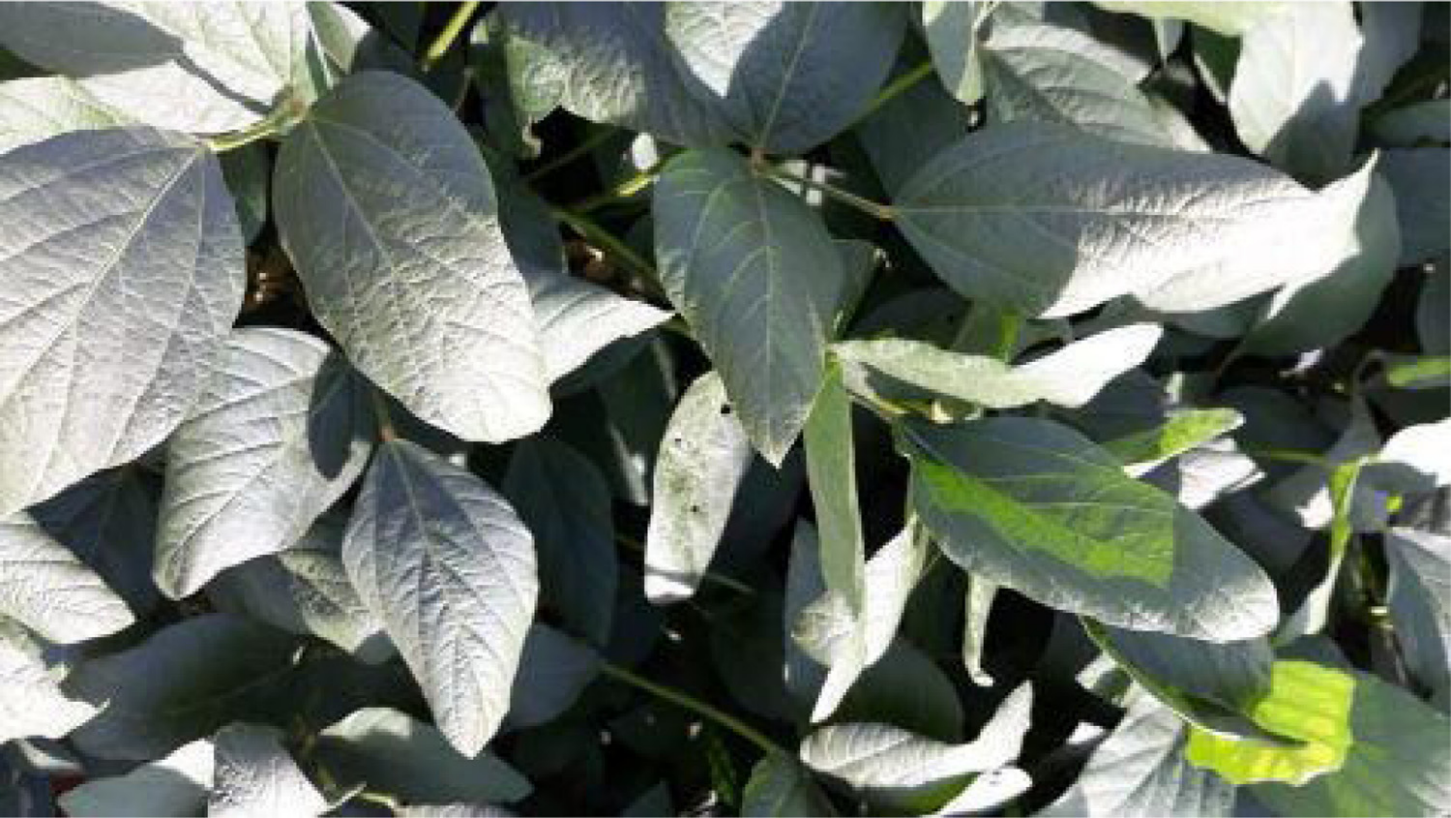
Sample of leaves affected by *Diabrotica speciosa.*

**Fig. 4:**
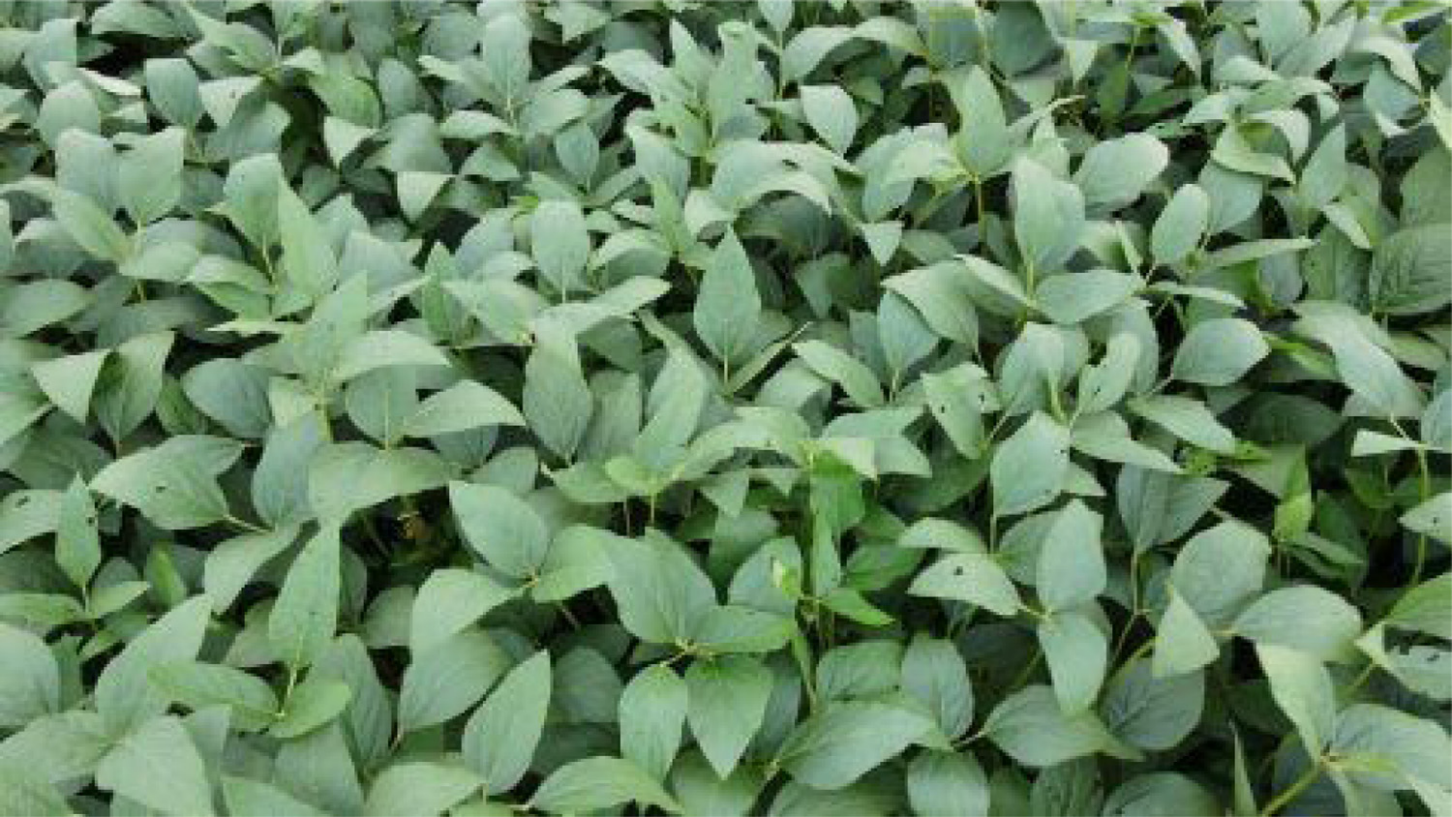
Another sample of leaves affected by *Diabrotica speciosa.*

## 3 Experimental Design, Materials and Methods

The images were captured in January 2021 in the central region of the state of Mato Grosso - Brazil, in several rural properties at the cities Lucas do Rio Verde and Nova Mutum. The process of capturing the images occurred during this period because it is the window for planting soybeans in the region. The capture took place at various stages of the plants, from budding to the adult stage, but before starting the yellowing phase of the leaves. As for the environment, the images were collected in adverse conditions, such as cloudy, sunny, drizzle, cloud shadows, and windy weather. The height of image capture varied between 20 cm and 1 meter away from the plant.

The pre-processing of the images followed four phases:

1. **Data annotation:** this phase was conducted manually by three experts who analyzed and classified the images in three groups;
2. **Dataset Split:** this phase involved the organization of the files in folders, following the classification conducted during data annotation;
3. **Image size standardization:** in this phase, we changed the size of the images for a standard size of 500 × 500 pixels.
4. **Data augmentation:** in this phase, we increased the size and diversity of the dataset by generating variations of the images considering different viewing angles.

The first and second phases of pre-processing organized the files in three folders according to the classification of each image: (I) healthy plants, (II) plants affected by catterpillars, and (III) plants attacked by *Diabrotica Speciosa*.

The third phase, *i.e.* image size standardization, involved the implementation of a Phython script to change the size of the photographs. To standardize the size and dimension of the images, we used the Python programming language along with CV2 libraries [5]. The images were standardized to have the same size and dimension. We used the Flipper technique to rotate the image in situations where the width to height rate of the image was less than 1. In this case, the script rotates the image by 90${}^{\circ }$ counterclockwise over the X-axis. This rotation allows a better display of the image concerning the foliage, allowing the identification of which ones are rectangular [6].

**Fig. 5:**
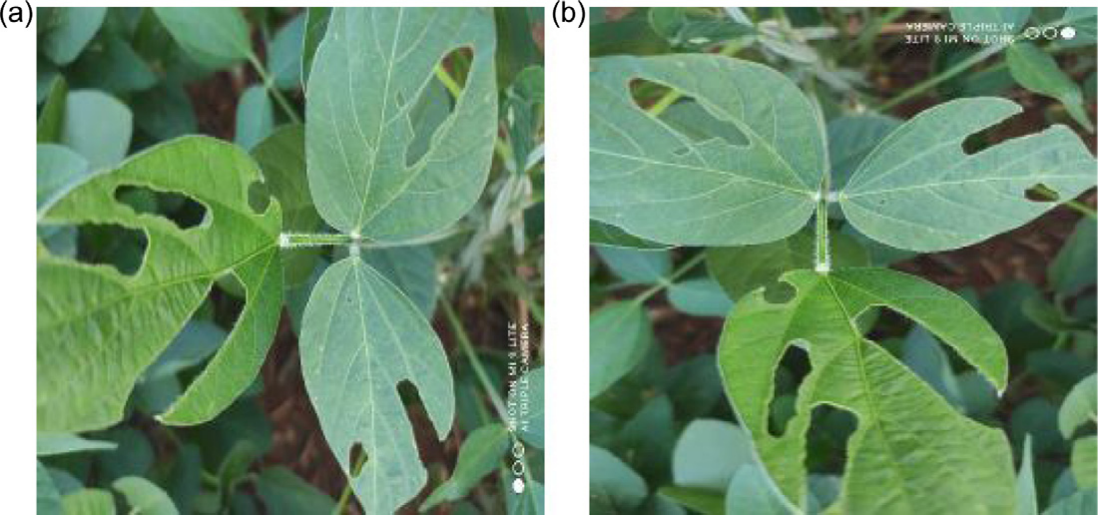
(a) Original caterpillar image. (b) Left rotated version of the same image.

In the fourth phase of pre-processing, we conduct a data augmentation procedure to improve the dataset both in terms of quantity of images and variability of angles available to train machine learning models [6]. Three rotation angles are considered: 90${}^{\circ }$ over the X-axis, 90${}^{\circ }$ over the Y-axis, and 180${}^{\circ }$ over the Y-axis. Fig. 5 (a) shows an example of an original image representing leaves attacked by caterpillars, and Fig. 5 (b) displays the same image after applying a 90${}^{\circ }$ rotation.

## Ethics Statement

We the authors assure consciously that for the article “Soybean Images Dataset for Caterpillar and *Diabrotica speciosa* pest detection and classification” the following is fulfilled:

1. This article is the authors’ own original work, which has not been previously published elsewhere;
2. The article is not currently being considered for publication elsewhere;
3. The article reflects the authors’ own research and analysis in a truthful and complete manner;
4. The article properly credits the meaningful contributions of co-authors;
5. All authors have been personally and actively involved in substantial work leading to the article, and will take public responsibility for its content.

We agree with the above statements and declare that this submission follows the policies of Solid State Ionics as outlined in the Guide for Authors and in the Ethical Statement.

## CRediT Author Statement

**Maria Eloisa Mignoni:** Writing the original article, image capture, publication of the dataset. **Aislan Honorato:** Image rotation and scaling script. **Rafael Kunst:** Review and writing. **Rodrigo Righi and Angelica Massuquitti:** Essay review and tips.

## Declaration of Competing Interest

The authors declare that they have no known competing financial interests or personal relationships which have, or could be perceived to have, influenced the work reported in this article.
